# Diagnostic Pitfall in Genomic Era: Discordant *SMARCA2* Finding in Patient with Megalencephalic Leukoencephalopathy with Subcortical Cysts

**DOI:** 10.3390/diagnostics16152314

**Published:** 2026-07-23

**Authors:** Chih-Hao Wang, Shuan-Pei Lin, Jon-Kway Huang, Nan-Chang Chiu

**Affiliations:** 1 Department of Pediatrics, MacKay Children’s Hospital, Taipei 104217, Taiwan; mmh4972@gmail.com; 2 Department of Pediatrics, MacKay Memorial Hospital, Taipei 104217, Taiwan; 4535lin@gmail.com; 3 School of Medicine, College of Medicine, MacKay Medical University, New Taipei City 252005, Taiwan; 4 International Rare Disease Center, MacKay Memorial Hospital, Taipei 104217, Taiwan; 5 Division of Genetics and Metabolism, Department of Medical Research, MacKay Memorial Hospital, Taipei 104217, Taiwan; 6 Department of Infant and Child Care, National Taipei University of Nursing and Health Sciences, Taipei 108306, Taiwan; 7 Department of Radiology, MacKay Memorial Hospital, Taipei 104217, Taiwan; johnmmh@gmail.com

**Keywords:** megalencephalic leukoencephalopathy with subcortical cysts, *SMARCA2*, leukodystrophy, neurodevelopmental disorder, magnetic resonance imaging, whole-exome sequencing

## Abstract

Next-generation sequencing has transformed the diagnostic approach to rare neurogenetic disorders. However, interpretation of molecular findings remains challenging when identified variants are discordant with the clinical phenotype. We report the case of a preschool-aged girl presenting with seizures, infantile-onset macrocephaly, and the characteristic magnetic resonance imaging (MRI) pattern of megalencephalic leukoencephalopathy with subcortical cysts (MLC). Whole-exome sequencing identified no pathogenic variants in the currently recognized MLC-associated genes. Instead, it detected a heterozygous *SMARCA2* splice-site variant that was classified as “likely pathogenic” by the testing laboratory according to the American College of Medical Genetics and Genomics criteria. However, the patient’s overall clinical presentation was not consistent with the currently recognized spectrum of *SMARCA2*-related disorders. Comprehensive genotype–phenotype correlation together with a two-year follow-up ultimately supported a diagnosis of classic MLC despite the discordant *SMARCA2* finding. This case highlights that molecular findings should complement, rather than override, established clinicoradiological evidence, particularly in disorders with highly characteristic imaging phenotypes.

**Figure 1:**
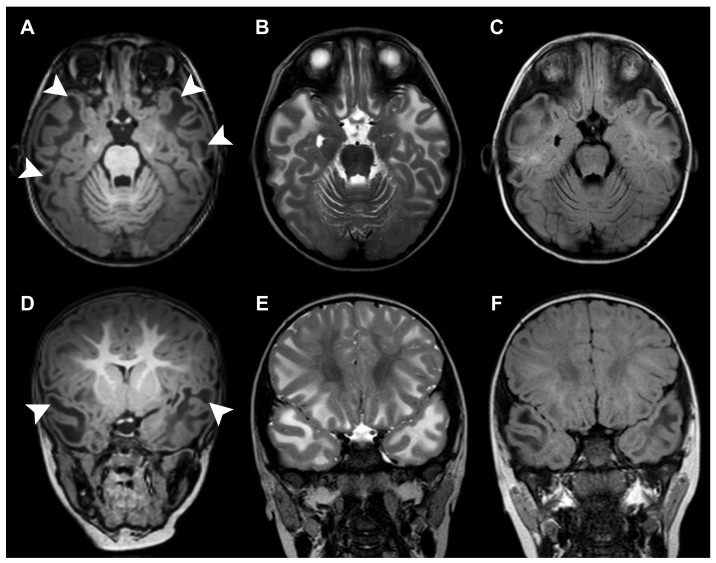
Brain magnetic resonance imaging (MRI) demonstrating a classic pattern of megalencephalic leukoencephalopathy with subcortical cysts (MLC). A 3-year-6-month-old girl with a history of febrile seizures and global developmental delay receiving rehabilitation was admitted because of a complex febrile seizure characterized by lateral gaze deviation and clonic movements lasting approximately 5 min. There was no family history of neurological or neurodevelopmental disorders. She had progressive macrocephaly since 18 months of age, with a head circumference of 53.7 cm (>97th percentile). Physical and neurological examinations revealed no focal deficits, and routine laboratory investigations were unremarkable. Electroencephalography revealed no epileptiform discharges. Brain MRI was performed. Axial (**A**–**C**) and coronal (**D**–**F**) brain MRI images are shown. T1-weighted images (**A**,**D**) demonstrate multiple hypointense cystic lesions in the subcortical white matter of the bilateral anterior temporal regions (arrowheads). Corresponding T2-weighted images (**B**,**E**) reveal extensive hyperintensity of the supratentorial white matter with a characteristic swollen appearance, predominantly involving the subcortical regions. On T2-weighted fluid-attenuated inversion recovery (FLAIR) images (**C**,**F**), the cystic components show relative signal suppression with surrounding white-matter hyperintensity. The overall imaging appearance, characterized by extensive supratentorial white-matter involvement with subcortical cysts most prominent in the anterior temporal lobes, is highly characteristic of MLC [1].

**Figure 2:**
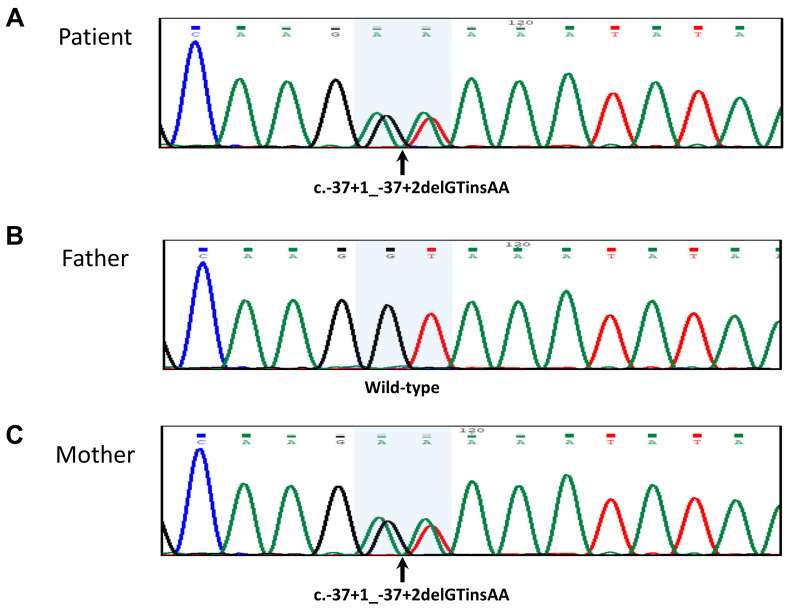
Sanger sequencing confirmation of the *SMARCA2* variant. Given the early-onset seizures, developmental delay, macrocephaly, and characteristic MLC neuroimaging findings, metabolic and genetic evaluations were performed. Metabolic screening was unremarkable. Whole-exome sequencing with mitochondrial genome analysis found no pathogenic variants in known MLC-associated genes (*MLC1*, *HEPACAM*, *GPRC5B*, and *AQP4*) [2,3]. Instead, a heterozygous splice-site variant in *SMARCA2* (NM_003070.5: c.-37+1_-37+2delGTinsAA) was identified and classified as likely pathogenic in the initial laboratory report based on automated American College of Medical Genetics and Genomics (ACMG) variant interpretation [4]. Subsequent Sanger sequencing confirmed the presence of the heterozygous *SMARCA2* variant in both the patient (**A**) and her asymptomatic mother (**C**), whereas the father (**B**) carried the wild-type allele. *SMARCA2*-related neurodevelopmental disorders and MLC are distinct with different genetic etiologies and pathophysiological mechanisms. While MLC is a leukodystrophy linked to ion and water homeostasis [3], *SMARCA2*-related disorders arise from defects in the ATP-dependent chromatin remodeling complex [5]. Both conditions can present with nonspecific features like epilepsy and developmental delay but do not share a known pathogenesis [3,5]. The diagnosis of MLC is primarily based on its characteristic clinical and neuroradiological features, including infantile-onset macrocephaly, seizures, and diffused swollen white matter with subcortical cysts, mostly within the anterior temporal lobes [2]. The primary role of molecular genetic testing is to further subclassify patients with a clinical diagnosis of MLC into either the “classic” phenotype (caused by biallelic variants in *MLC1* or *HEPACAM*, or a heterozygous variant in *GPRC5B*) or the “improving” phenotype (caused by a heterozygous variant in *HEPACAM* or biallelic variants in *AQP4*), thereby facilitating genetic counseling. Notably, in a small proportion of patients, no causative pathogenic variants can be identified. Even in such cases, the clinical diagnosis of MLC can be firmly established based on typical clinical manifestations and characteristic neuroimaging hallmarks, as no other known disorders share this highly specific MRI pattern [3]. Previous reports have likewise established the diagnosis based on these clinical and MRI features, occasionally without genetic confirmation [6,7,8]. Accordingly, these MRI findings are often considered pathognomonic for MLC [7]. However, our case illustrates an important diagnostic challenge. The patient exhibited classic clinicoradiological features of MLC but harbored a heterozygous *SMARCA2* variant rather than pathogenic variants in known MLC-associated genes. Pathogenic *SMARCA2* variants are associated with neurodevelopmental disorders including Nicolaides–Baraitser syndrome (NCBRS) and blepharophimosis–intellectual disability syndrome [5,9]. Despite the variant being initially reported as “likely pathogenic” in the laboratory report, rigorous genotype–phenotype correlation revealed substantial discordance. Several lines of evidence strongly argue against this variant being responsible for our patient’s phenotype. First, the variant is located outside the ATPase domain, whereas established disease-causing variants cluster almost exclusively within the C-terminal helicase region of the ATPase domain (exons 15–25) [5]. Second, the splice-site variant is predicted to cause loss of function, a feature that may have contributed to its classification as “likely pathogenic” under the laboratory’s automated ACMG-based interpretation. However, current evidence indicates that NCBRS arises through dominant-negative or gain-of-function mechanisms rather than simple loss of function [10]. Furthermore, animal studies have shown that mice lacking functional *Smarca2* do not exhibit major developmental abnormalities [11], supporting the concept that haploinsufficiency alone is unlikely to cause *SMARCA2*-related disease. Third, the variant was inherited from an unaffected mother, whereas pathogenic *SMARCA2* variants are typically de novo. Moreover, *SMARCA2*-related disorders are not known to exhibit reduced penetrance [5]; therefore, the presence of a truly pathogenic heterozygous variant in an asymptomatic parent would be unexpected. Finally, a distinct contrast is evident in their expected neuroimaging features. The patient exhibited progressive macrocephaly and the highly specific MRI pattern of MLC, characterized by extensive swollen supratentorial white matter and prominent subcortical cysts, particularly in the anterior temporal lobes [1,2]. In stark contrast, patients with pathogenic *SMARCA2* variants typically present with acquired microcephaly and generally inconspicuous or nonspecific brain MRI findings (e.g., mostly normal imaging without structural defects) [5,12], completely lacking the cystic leukoencephalopathy features observed in our patient. Collectively, these lines of evidence indicate that the identified *SMARCA2* variant is unlikely to explain the patient’s phenotype and should not supersede the clinicoradiological diagnosis of MLC. Returning to our patient, a two-year longitudinal follow-up demonstrated recurrent afebrile seizures and gait ataxia, consistent with the expected clinical evolution of classic MLC. In addition, clinical evaluations revealed severe intellectual disability and expressive language delay without apparent dysarthria. This clinical course during follow-up further reinforced the clinicoradiological diagnosis. It also highlights the enduring diagnostic value of the characteristic MRI pattern of MLC when potentially discordant genomic findings are interpreted. A limitation of our report is that we did not perform whole-genome sequencing (WGS). Although WES identified no pathogenic variants in known MLC-associated genes, this method retains inherent limitations in detecting deep intronic or large structural variants. Therefore, we could not completely exclude the possibility that such hidden variants exist in the MLC-related genes (e.g., *MLC1* or *HEPACAM*) of our patient. In unresolved cases where the clinical and radiological phenotypes strongly indicate a specific disorder like MLC, WGS should be considered as a crucial future diagnostic approach to uncover these potential non-coding variants.

## Data Availability

No new data were created or analyzed in this study. Data sharing is not applicable to this article.

## Abbreviations

The following abbreviations are used in this manuscript:

ACMG	American College of Medical Genetics and Genomics
FLAIR	Fluid-attenuated inversion recovery
MLC	Megalencephalic leukoencephalopathy with subcortical cysts
MRI	Magnetic resonance imaging
NCBRS	Nicolaides–Baraitser syndrome
WES	Whole-exome sequencing
WGS	Whole-genome sequencing
